# Diagnostic Algorithm for Secondary Extramammary Paget Disease from Institutional Cases and Literature Review

**DOI:** 10.3390/cancers17244014

**Published:** 2025-12-17

**Authors:** Salin Kiratikanon, Ayaka Fukui, Masahiro Hirata, Jakob M. T. Moran, Masakazu Fujimoto, Mai P. Hoang

**Affiliations:** 1 Department of Pathology, Massachusetts General Hospital, Boston, MA 02114, USA; s.kiratikanon@gmail.com (S.K.); jmoran0@mgb.org (J.M.T.M.); 2 Division of Dermatology, Department of Internal Medicine, Faculty of Medicine, Chiang Mai University, Chiang Mai 50200, Thailand; 3 Department of Diagnostic Pathology, Kyoto University Hospital, Kyoto 606-8507, Japan; fukui_a@kuhp.kyoto-u.ac.jp (A.F.); hiratama@kuhp.kyoto-u.ac.jp (M.H.); fujimasa@kuhp.kyoto-u.ac.jp (M.F.); 4 Department of Pathology, Harvard Medical School, Boston, MA 02115, USA

**Keywords:** extramammary Paget disease, secondary, TRPS1, SATB2, immunohistochemistry, GATA3, CDX2

## Abstract

**Background/Objectives**: Although clinicopathologic correlation with integration of clinical and radiographic data is the gold standard in distinguishing primary extramammary Paget disease (EMPD) from secondary EMPD, immunoprofiling of EMPD tumors enables distinction between primary and secondary EMPD. **Methods**: We evaluated the immunoprofiles of previously published cases in the literature as well as 12 secondary EMPD cases from our archives in order to construct a diagnostic algorithm that enables the distinction between primary and secondary EMPD. **Results**: Immunoprofiles of 480 primary (published cases) and 132 secondary (120 published cases and 12 institutional cases) EMPD cases were compared. CK7, CK20, CDX2, GATA3, GCDFP15, TRPS1, and SATB2 expression was significantly different in primary EMPD versus colonic secondary EMPD (*p* < 0.001 for all except SATB2, *p* = 0.036). CK20, GCDFP15, TRPS1, p63 and uroplakin II/III expression was significantly different in primary EMPD versus urothelial secondary EMPD (*p* < 0.001). CK7, CDX2, SATB2, GATA3 and p63 expression was significantly different in colonic versus urothelial secondary EMPD. CK20, CDX2, and GCDFP15 expression was significantly different in colonic versus prostatic secondary EMPD. CK20 expression was significantly different in colonic versus prostatic secondary EMPD (*p* = 0.018). CK20, GCDFP15 and TRPS1 are helpful in the distinction of primary EMPD versus colonic and urothelial secondary EMPD (*p* < 0.001). **Conclusions**: We propose that the initial IHC panel should include TRPS1, CK7 and CK20. In TRPS1-negative cases, additional immunostains should be performed: CDX2 and SATB2 for colonic; p63, GATA3 and uroplakin II/III for urothelial; and PSA and NKX3.1 for prostatic secondary EMPD.

## Simple Summary

Distinguishing between primary and secondary extramammary Paget disease (EMPD) is crucial as the underlying causes and treatments differ. Although radiographic and clinical correlation is essential, immunohistochemistry (IHC) enables a quick and practical method of distinguishing primary EMPD from secondary EMPD. We evaluated 480 primary EMPD cases and 132 secondary EMPD cases (from a literature review and from our institutional cases). Primary EMPD typically expresses CK7 and TRPS1. Colonic secondary EMPD usually expresses CK20, CDX2, and SATB2, and urothelial secondary EMPD demonstrates p63, GATA3, and uroplakin II/III expression. Based on these IHC profiles, we propose that the initial IHC panel should include TRPS1, CK7 and CK20. In TRPS1-negative cases, additional immunostains should be performed: CDX2 and SATB2 for colonic secondary EMPD; p63, GATA3 and uroplakin II/III for urothelial secondary EMPD; and PSA and NKX3.1 for prostatic secondary EMPD. This IHC algorithm allows pathologists to distinguish primary EMPD from secondary EMPD and guide clinical management.

## 1. Introduction

Extramammary Paget’s disease (EMPD) is a rare intraepidermal cutaneous adenocarcinoma, that typically arises in apocrine-rich skin such as the genitalia, perineum, perianal area, and axilla [1,2,3]. Despite its relatively indolent course, EMPD carries significant clinical impact owing to its potential association with synchronous or metachronous internal malignancies and its tendency for local recurrence, posing challenges in achieving timely and accurate diagnosis [3,4].

EMPD can be broadly classified into primary and secondary forms [3]. Primary EMPD is thought to arise from intraepidermal malignant transformation of a cell in underlying adnexal structures. Secondary EMPD results from epidermotropic spread of an underlying visceral carcinoma, most commonly of anorectal or urothelial origin [3,5,6]. While primary EMPD can be managed with local excision or with non-surgical modalities [7], secondary EMPD typically reflects advanced disease with poorer outcomes and prognosis is dependent on treatment of the underlying visceral carcinoma [8,9]. Distinction between primary and secondary EMPD is crucial for clinical management, prognosis, and therapeutic decision-making. Notably, secondary EMPD has been reported in approximately 10.8–18.1% of cases in prior cohorts [1,8,10].

Given the importance of distinguishing primary and secondary EMPD, the distinct IHC profiles of primary and secondary EMPD is of clinical utility. Conventional IHC panels including keratin 7 (CK7), keratin 20 (CK20), gross cystic disease fluid protein 15 (GCDFP15), caudal-type homeobox 2 (CDX2), uroplakin II/III and GATA binding protein 3 (GATA3) are routinely employed to distinguish primary and secondary EMPD; however, overlapping immunoprofiles can complicate interpretation and contribute to diagnostic uncertainty [3,5,8]. Of note, trichorhinophalangeal syndrome type 1 (TRPS1) has emerged as a sensitive marker for primary EMPD, further refining the diagnostic approach [11].

Although clinicopathologic correlation with integration of clinical and radiographic data is the gold standard in distinguishing primary EMPD from secondary EMPD, immunoprofiling of EMPD tumors enables distinction between primary and secondary EMPD. For these reasons, several immunostains and a systematic panel-based approach are needed to improve diagnostic accuracy. In our study, we aim to characterize the clinical, histopathologic, and immunohistochemical parameters of EMPD. We collected the immunohistochemical profiles of previously published cohorts of primary EMPD and secondary EMPD. We also included an additional cohort of secondary EMPD from two university archives. We constructed a diagnostic algorithm from our cases and previously published data that enables pathologists to distinguish primary and secondary EMPD (of colonic, urothelial and prostatic origin).

## 2. Materials and Methods

### 2.1. Study Design and Data Collection

The study has been approved by the institutional review board at both institutions. We retrospectively searched the pathology files of Massachusetts General Hospital (MGH), Boston, MA, USA, and Kyoto University Hospital (KUH), Kyoto, Japan from 1990 to 2025 for secondary EMPD cases. We followed The Strengthening the Reporting of Observational Studies in Epidemiology (STROBE) [12] reporting guidelines.

Secondary EMPD was defined as EMPD with synchronous (occurred within 1 year before or after EMPD diagnosis) or metachronous internal malignancies up to 10 years before or after EMPD diagnosis [6,10]. Published associated internal malignancies were colorectal and anorectal carcinoma, male and female genital tract cancers and urinary bladder cancers [6,10].

Inclusion criterion for secondary EMPD was patients who had been diagnosed with secondary EMPD with confirmed histological diagnosis, supporting IHC studies and associated internal malignancy. The cases were reviewed by the contributing pathologists and the corresponding author. Discrepancy was resolved via virtual consensus. We excluded cases without IHC studies or with uncertain diagnosis. The patient’s age and gender, lesion morphology, location, histological features, immunoprofiles, date of diagnosis, date of recurrence, date of metastasis, date of last follow-up, and clinical and radiographic investigations to work up for internal malignancies were recorded from clinical chart reviews.

### 2.2. Immunohistochemistry

Immunohistochemical studies were performed on 5-micrometer-thick tissue sections using a Bond 3 automated immunostainer (Leica Microsystems, Bannockburn, IL, USA), with primary antibodies against CDX2 (undiluted, EPR2764Y, Cell Marque, Rocklin, CA, USA), GATA3 (undiluted, L50-823, Leica Microsystems), GCDFP15 (undiluted, 23a3, Leica Microsystems), CK7 (RN7, predilute, Leica Microsystems), CK20 (ks20.8, prediluted, Leica Microsystems), NK3 Homeobox 1 (NKX3.1) (undiluted, ep356, BioSB, Goleta, CA, USA), p63 (undiluted, 4A4, Roche, Basel, Switzerland), Special AT-rich sequence-binding protein 2 (SATB2) (undiluted, cl0276, Cell Marque), TRPS1 (undiluted, EP392, BioSB), and uroplakin II/III (undiluted, BC21/BC17, BioCare, Tempe, AZ, USA). Heat-induced epitope retrieval with EDTA-based pH9 epitope retrieval solution (Leica Microsystems) was performed for 30 min for uroplakin II/III and 20 min for remaining antibodies. Polymer-based detection was used. Positive and negative controls were included in each IHC run. Positivity was defined as staining greater than 10% of the tumor cells.

### 2.3. Statistical Analyses

Categorical variables were presented as counts and percentages, while continuous variables were presented as mean ± standard deviations (SDs) or median and interquartile range (IQR). Overall comparisons among EMPD subgroups were performed using Pearson’s chi-square or Fisher’s exact test, as appropriate. Subsequently, pairwise comparisons with Bonferroni correction were applied to identify significantly different subgroup pairs. Data analyses were performed using the STATA MP 16.0 (STATA Corp, College Station, TX, USA). A two-tailed *p*-value < 0.05 was considered to be statistically significant.

### 2.4. Literature Review

**Figure 1:**
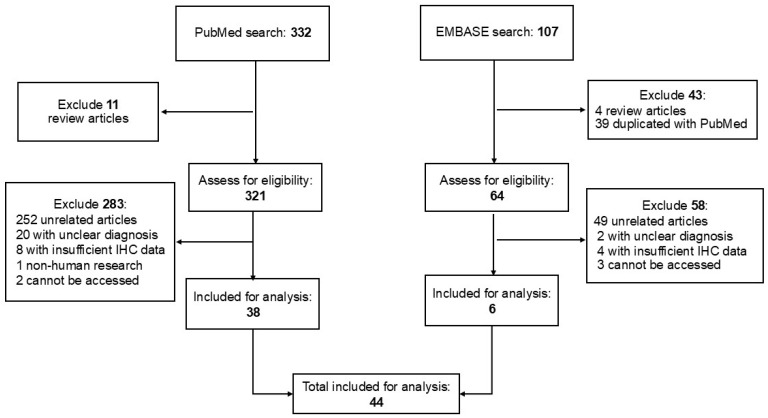
Summary of the literature search and study selection.

To identify primary and secondary EMPD previously described with available IHC results, PubMed and Excerpta Medica dataBASE (EMBASE) searches were conducted using the following combination of search terms: (secondary OR scrotal OR vulvar OR genital OR perianal) AND (extramammary Paget disease OR extramammary Paget’s disease OR extramammary Paget disease OR extramammary, Paget disease) AND (immunohistochemistry OR immunohistochemical OR markers OR expression). We applied filters to include studies published from 1990 to the search date in August 2025, written in English, and involving human subjects. The full search strategy and results are presented in Figure 1. Published reports from January 1990 to August 2025 with IHC findings in primary EMPD and secondary EMPD were included for statistical analyses (Figure 1). While both case reports and studies were included for secondary EMPD, case reports of primary EMPD were excluded. The titles and abstracts were reviewed to select the applicable manuscripts with available IHC results. The selected manuscripts were further reviewed in depth. To facilitate statistical analyses, we recorded the published IHC results as positive versus negative. Studies in the literature review that did not clearly specify whether the cases were primary or secondary EMPD were excluded.

## 3. Results

### 3.1. Clinical and Histopathologic Data of 12 Secondary EMPD at Two Institutions

**Figure 2:**
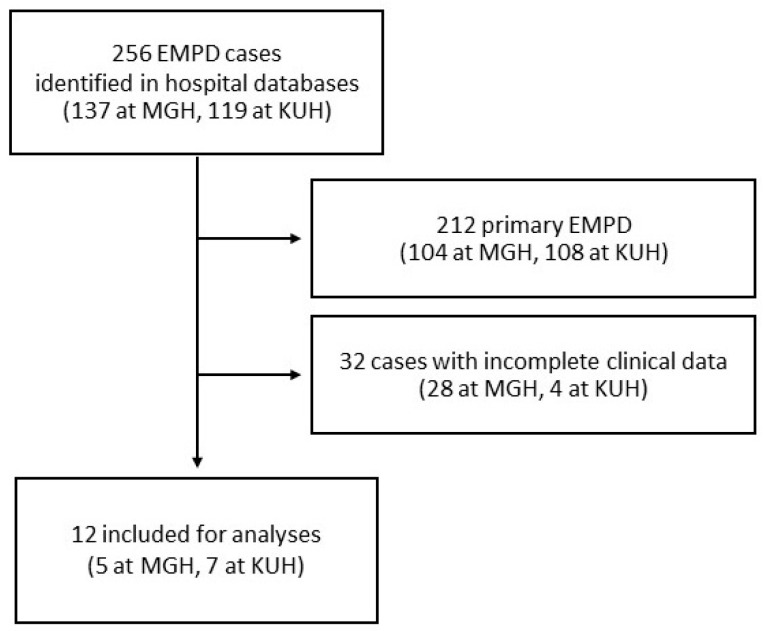
Flow chart of case inclusion at Massachusetts General Hospital (MGH) and Kyoto University Hospital (KUH).

Upon searching the MGH and KUH databases from 1990 to 2025, a total of 256 patients diagnosed with EMPD, with or without available archival tissues, were identified. Of these, 212 cases of primary EMPD and 32 cases with incomplete clinical data were excluded. Finally, 12 patients with secondary EMPD were included in the study (Figure 2). All patients with available clinical data had undergone, at the discretion of the clinical team, at least one of the following clinical tests for evaluation for occult malignancies: colonoscopy, cystoscopy, imaging studies, and serum tumor markers.

The clinical characteristics of 12 patients with secondary EMPD are summarized in Table 1. Seven patients were male (58.3%) and five were female (41.7%), with a mean age of 71.9 ± 9.3 years. The anal/perianal region was the most affected site (6/12, 50%), followed by the vulva, penis, perineum, and other locations. Notably, one patient had lesions on multiple locations including vulva, perineum, and anal/perianal region. Colorectal carcinoma was the most frequently associated internal malignancy (7/12, 58.3%), followed by urothelial carcinoma of the bladder (5/12, 41.7%). No cases of secondary EMPD associated with prostate cancer were identified in our cohort. All internal malignancies were confirmed histologically. The interval between EMPD diagnosis and detection of the primary internal malignancy ranged from 84 months before to 109 months after cancer diagnosis (median 0 months; IQR-4.5–31 months). The median follow-up duration from EMPD diagnosis to the last date of follow-up was 45.5 months (IQR 23.5–122.5), during which six patients experienced EMPD recurrence.

**Figure 3:**
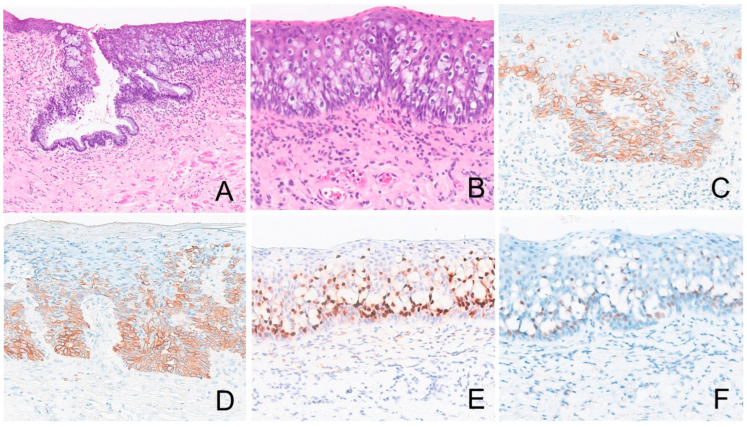
Extramammary Paget disease of colorectal origin. The tumor ((**A**), 100×, (**B**), 200×) is positive for CK7 ((**C**), 200×), CK20 ((**D**), 200×), CDX2 ((**E**), 200×), and SATB2 ((**F**), 200×).

**Figure 4:**
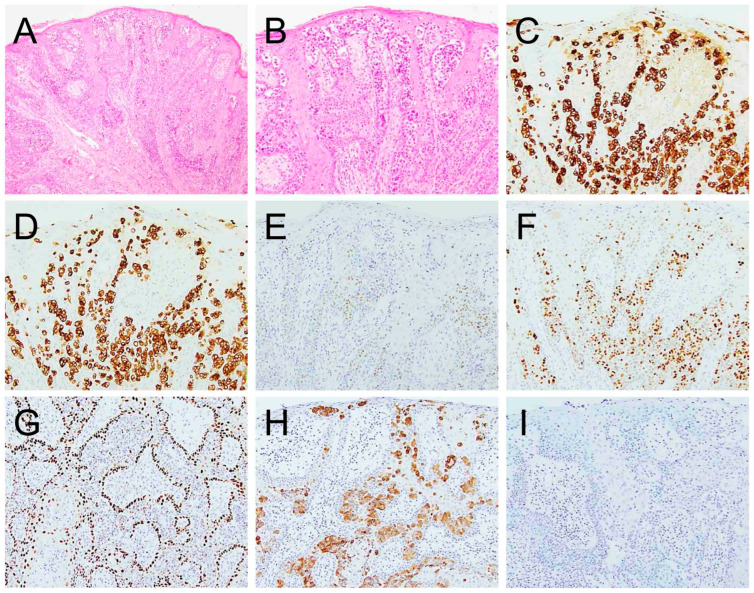
Extramammary Paget disease of urothelial origin ((**A**), 100×, (**B**), 200×) is positive for CK7 ((**C**), 200×), CK20 ((**D**), 200×), CDX2 ((**E**), 200×), GATA3 ((**F**), 200×), p63 weakly ((**G**), 200×), and uroplakin II/III ((**H**), 200×). It is negative for TRPS1 ((**I**), 200×).

Table 2 summarizes the IHC profiles of each patient. All colorectal secondary EMPD cases demonstrated positive staining for CK20 and CDX2. SATB2 was positive in three of four cases (75%) and CK7 was positive in five of seven cases (71.4%) whereas GCDFP15, p63, TRPS1, and GATA3 were negative in all cases (Figure 3). In the urothelial secondary EMPD group, CK7, CK20, GATA3, and uroplakin II/III were positive in all tested cases. P63 and CDX2, were positive in four of five (80%), 3 of 5 (60%) cases, respectively (Figure 4). GCDFP15, NKX3.1, SATB2, and TRPS1 were consistently negative across all patients.

### 3.2. Comparison of Primary Versus Secondary EMPD

In our literature review, 480 primary EMPD and 120 secondary EMPD cases (79 colonic, 36 urothelial, and 5 prostatic) with immunohistochemical results were identified. When combined with the 12 cases from our institutional cohort, a total of 132 secondary EMPD cases (86 colonic, 41 urothelial, and 5 prostatic) were included for analysis. Table 3 summarizes the IHC profiles of primary EMPD in comparison with secondary EMPD of colonic, urothelial, and prostatic origins.

**Figure 5:**
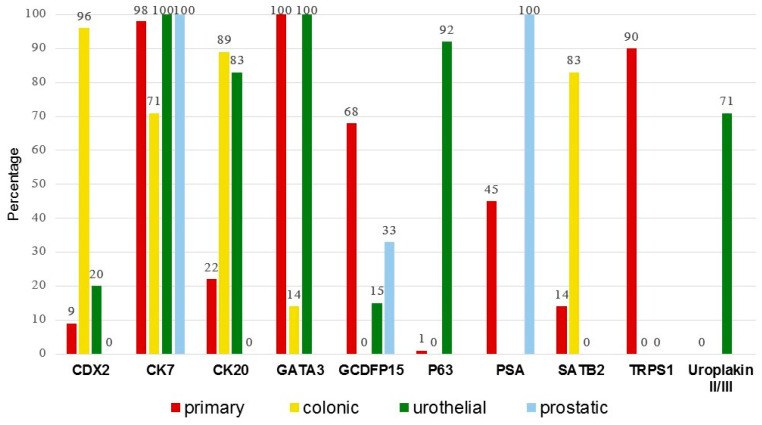
Percentage of IHC positivity across subgroups. Of the 132 secondary extramammary Paget disease cases, 120 are from the literature and 12 are from the current cohort.

Significant differences in the IHC marker expression among EMPD subgroups (primary, colonic, urothelial, and prostatic secondary EMPD) were observed for CDX2 (*p* < 0.001), CK7 (*p* < 0.001), CK20 (*p* < 0.001), GATA3 (*p* < 0.001), GCDFP15 (*p* < 0.001), p63 (*p* < 0.001), SATB2 (0.001), TRPS1 (*p* < 0.001), and uroplakin II/III (0.001) (Table 3). Detailed datasets derived from the literature review and the current cohort are presented in Supplementary Table S1. Figure 5 illustrates the IHC positivity rates across primary, colonic, urothelial, and prostatic EMPD subgroups.

Further pairwise comparisons between each EMPD subgroup highlight significant differences in expression of CK7, CK20, CDX2, GATA3, GCDFP15, p63, SATB2, TRPS1, and uroplakin II/III (Table 4). CK7, CK20, CDX2, GATA3, GCDFP15, TRPS1, and SATB2 expression is significantly different in primary EMPD versus colonic secondary EMPD (*p* < 0.001 for all except SATB2, *p* = 0.036). CK20, GCDFP15, TRPS1, p63 and uroplakin II/III expression is significantly different in primary EMPD versus urothelial secondary EMPD (*p* < 0.001). CK7, CDX2, SATB2, GATA3 and p63 expression are significantly different in colonic versus urothelial secondary EMPD. CK20 and CDX2 expression are significantly different in colonic versus prostatic secondary EMPD. CK20, GCDFP15 and TRPS1 are helpful in the distinction of primary EMPD versus colonic and urothelial secondary EMPD (*p* < 0.001).

## 4. Discussion

We report the clinical and histopathologic parameters of a cohort of 12 secondary EMPD patients along with previously published data of primary and secondary EMPD cases (total of 612 EMPD cases). This pooled analysis represents the largest dataset to date enabling comprehensive evaluation of the immunoprofiles of primary and secondary EMPD subtypes.

Given the overlapping immunoprofiles of CK7, CK20 and CDX2 in primary and secondary EMPD, additional immunostains are needed to distinguish between the two. Furthermore, the overlapping immunoprofiles highlight the need for a panel-based approach rather than reliance on a single marker to accurately distinguish primary from secondary EMPD. CK7 expression can be seen in 81% of secondary EMPD, while CK20 and CDX2 can be seen in 22% and 9% of primary EMPD, respectively (Table 3). The sensitivity of GCDFP-15 for primary EMPD is only 68%. In addition, GCDFP-15 expression can be seen in 5% of secondary EMPD cases (Table 3). Overlapping expression was also noted for CAM5.2, carcinoembryonic antigen (CEA), epithelial membrane antigen (EMA), HER2, cyclin D1, Mucin 1 (MUC1), and MUC2. No expression of CD15, CK1, CK5/6, CK10, CK13, CK14, CK15, estrogen receptor (ER), lysozyme, and S100 was seen in both primary and secondary EMPD (Table 3). Since IHC for p40, B72.3, p53, programmed death-ligand 1 (PD-L1), and RANKL was only performed on primary EMPD and IHC for CK8, CK18, CK19, MUC5AC, p16, MUC6, pan-keratin, progesterone receptor (PR), thrombomodulin, and Wilm’s tumor 1 (WT1) were only performed on secondary EMPD; the diagnostic role of these stains in distinguishing primary versus secondary EMPD cannot be evaluated (Table 3).

### 4.1. TRPS1 Expression in Primary and Secondary EMPD

TRPS1 has been recognized as a sensitive marker for tumors of breast origin [11] and mesenchymal tumors arising in breast parenchyma [58]. TRPS1 expression has also been observed in a variety of tumors including cutaneous adnexal tumors, squamous cell carcinoma, and mammary Paget disease [59]. Recently TRPS1 has been reported as a useful diagnostic marker in the setting of EMPD, as it is positive in primary EPMD and negative in secondary EMPD [13,14]. TRPS1 was expressed in 90% of primary EMPD cases and was consistently negative in secondary EMPD [13,14]. Previous studies also reported 100% TRPS1 positivity in primary vulvar and scrotal EMPD [14]; however, lack of TRPS1 expression has been observed in perianal lesions [13,14]. These findings highlight the diagnostic utility of TRPS1 in confirming primary EMPD and excluding secondary EMPD.

### 4.2. SATB2, CK20 and CDX2 Expression in Colonic Secondary EMPD

SATB2, a DNA-binding protein involved in transcriptional regulation and chromatin remodeling, has been established as a sensitive marker for colorectal carcinoma, although it is not specific since positivity can be seen in gastrointestinal and pancreatobiliary adenocarcinoma [60,61]. Of note, SATB2 has been shown to be a more sensitive marker of colorectal origin than CDX2 in a study of 44 mucinous colorectal carcinomas and 175 non-colorectal mucinous tumors by Brettfeld et al. [62]. In the setting of mucinous carcinoma, SATB2 is a good diagnostic marker since secondary EMPD arising in association with mucinous carcinoma is typically of colorectal origin, and not from non-colorectal sites. SATB2 expression has also been described in other malignancies, including renal and urologic carcinomas, carcinoid tumors, and small intestinal neoplasms [63]. In our study, SATB2 was expressed in 10/12 (83%) perianal colonic secondary EMPD cases, 1/7 (14%) primary vulvar EMPD case with perianal involvement and none of the urothelial secondary EMPD cases. Although the overall positivity rate of CDX2 in colonic secondary EMPD exceeded that of SATB2 (96% vs. 83%) [20,24,25,26,27,29,31,40,42,44,47], CDX2 expression was observed in approximately 20% of urothelial secondary EMPD, whereas SATB2 remained consistently negative. Therefore, SATB2 may be particularly useful in differentiating perianal colorectal secondary EMPD from urothelial secondary EMPD with perianal involvement, especially in TRPS1-negative cases. Additional studies are needed to validate this diagnostic approach.

Based on the pooled analyses performed in our study, CDX2 and CK20 show overlapping expression in EMPD as outlined in Table 3. CK20 is positive in 22% of primary EMPD and CDX2 is positive in 20% of urothelial secondary EMPD. Colonic secondary EMPD is CK20-positive in 96% and CDX2-positive in 89% of cases. Urothelial secondary EMPD is CK20-positive in 83% of cases and CDX2-positive in 20% of cases. None of the prostatic secondary EMPD express CDX2 or CK20. These findings demonstrate that CDX2 is more sensitive (96% versus 89%) and more specific than CK20 in detecting colonic secondary EMPD.

### 4.3. GATA3, p63 and Uroplakin Expression in Urothelial Secondary EMPD

GATA3 is a member of the GATA family of transcription factors protein involved in embryogenesis, cell proliferation, and differentiation in multiple human tissues and organs, including breast, genitourinary system, parathyroid, skin, central nervous system, and hematopoietic systems [64,65,66,67]. It had been identified as a highly sensitive and specific marker for breast and urothelial carcinomas [68,69,70,71].

In primary EMPD, GATA3 demonstrates high sensitivity, reaching 100% in several series [72]; however, it lacks specificity, as it is also positive in secondary EMPD of urothelial origin [23]. Consistent with these reports, both our institutional cohort and prior studies [48,49,52,54] showed GATA3 expression in 100% of primary EMPD and urothelial secondary EMPD cases, indicating that GATA3 cannot reliably distinguish between these two entities. In contrast, adjunctive p63 staining may aid in this differential diagnosis. p63 positivity was observed in all eight urothelial secondary EMPD cases, but in only 1.4% of primary EMPD [15,16,17,18,19,20,21,22]. Notably, all previously studied lesions were located on genital sites (vulva, vagina, and glans penis), except for one case in our cohort involving the abdomen. Although uroplakin expression is significantly different in primary EMPD and urothelial secondary EMPD, expression of uroplakin in colorectal and prostatic secondary EMP has not been studied.

### 4.4. PSA and NKX3.1 Expression in Prostatic Secondary EMPD

**Figure 6:**
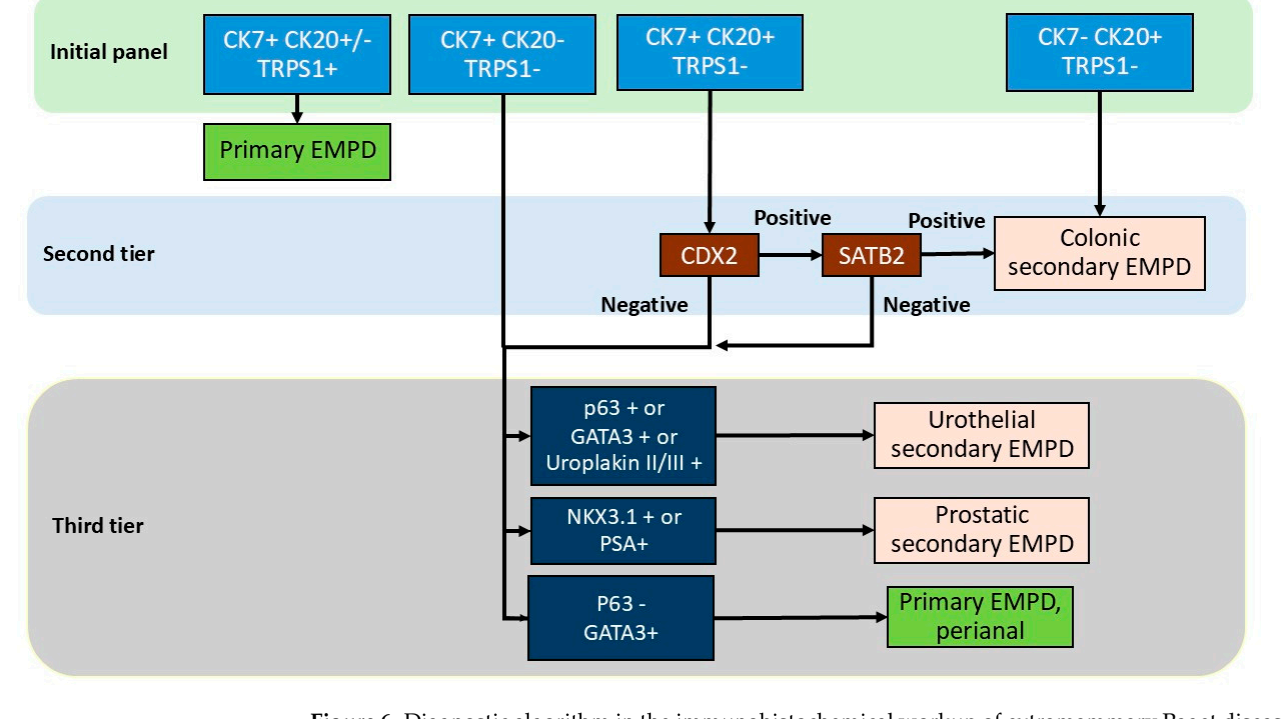
Diagnostic algorithm in the immunohistochemical workup of extramammary Paget disease.

Due to the rarity of prostatic secondary EMPD, available data remain limited. Prostatic-specific antigen (PSA) has been the most frequently tested marker, with expression reported in two cases of prostatic secondary EMPD [56,57]. However, its specificity is limited, as PSA expression has also been observed in up to 45% of primary EMPD cases (Table 3). This finding is consistent with another study in which all primary EMPD cases were PSA-positive, whereas none of the prostatic secondary EMPD cases showed reactivity [73]. NKX3.1 is a sensitive and specific marker for prostatic adenocarcinoma [74]; however, some studies have shown no reactivity in the rare prostatic secondary EMPD cases. Conversely, NKX3.1 expression has been detected in some genital primary EMPD cases. Therefore, these findings suggest that no single IHC marker can reliably distinguish prostatic secondary EMPD, underscoring the need for a comprehensive IHC panel including CK7, CK20, TRPS1, PSA, and NKX3.1 rather than reliance on a single immunostain (Figure 6).

### 4.5. Proposed Immunohistochemical Algorithm

Based on the collective data, we propose a practical stepwise IHC-based diagnostic algorithm for distinguishing primary from secondary EMPD subtypes. Initial evaluation with CK7, CK20, and TRPS1 is recommended. Positivity for both CK7 and TRPS1 supports a diagnosis of primary EMPD. Primary EMPD, especially perianal primary EMPD, can be TRPS1-negative in approximately 10% of cases; however, in these cases, CK7 is positive and CK20 is negative. In TRPS1-negative cases, additional markers including CDX2, SATB2, p63 and GATA3 are needed to exclude the possibility of colonic or urothelial secondary EMPD. Lack of CK7 and TRPS1 expression together with CK20 positivity are supportive of colonic secondary EMPD. CK7 and CK20 positivity together with TRPS1 negativity should prompt second-tier testing with CDX2 and SATB2. Positivity for SATB2 confirms colonic secondary EMPD. If CDX2 and SATB2 are both negative, or if CK7 is positive but CK20 and TRPS1 are negative, third-tier immunostains including p63, GATA3, uroplakin II/III, PSA, and NKX3.1 should be performed to further classify the tumor as urothelial or prostatic secondary EMPD. Notably, if p63 is negative with variable GATA3 or PSA expression, the results favor a primary perianal EMPD. Of note, p63 expression in primary EMPD may be more common in perianal lesions and that site-specific staining patterns should be considered in the algorithm. PSA staining should be interpreted with caution as well given its reported positivity in primary EMPD, and in the context of clinical and imaging findings, serum PSA level, and prostate biopsy result.

With respect to the effect of treatment effect on immunoprofile of EMPD, in a study of 412 post-treatment tissue samples from three women with primary perianal EMPD, topical chemotherapy induced cytologic atypia of epidermal keratinocytes, but did not alter the immunoprofile of EMPD [75]. Therefore, we suspect that our proposed algorithm is applicable to treated tumor as well; however, further studies performed on treated tumors are warranted. This diagnostic algorithm with a tier approach can provide a rational, accessible framework to guide pathologic diagnosis and subsequent clinical evaluation (Figure 6 and Figure S1).

### 4.6. Strength and Limitations

This pooled analysis represents the most extensive dataset of secondary EMPD to date. By integrating data from multiple reports and our cohort, we were able to provide a comprehensive overview of the IHC profiles of each EMPD subtype. The proposed algorithm offers a rapid and accessible diagnostic approach that can be employed by pathologists to facilitate targeted clinical workup. The main limitations include heterogeneity among included studies in defining secondary EMPD, variations in IHC techniques, and interpretative thresholds, which may account for some inconsistencies in reported results. To mitigate these effects, we applied strict inclusion criteria to ensure maximal data uniformity. Further studies are warranted to explore unresolved issues, including the expression of SATB2 in EMPD from non-perianal sites and the diagnostic performance of NKX3.1 in prostatic secondary EMPD.

## 5. Conclusions

We propose a diagnostic algorithm with a tiered immunohistochemical approach in order to distinguish primary from subtypes of secondary EMPD. The initial panel should include CK7, CK20, and TRPS1. Concurrent positivity for all three markers, or for CK7 and TRPS1 with CK20 negativity, supports a diagnosis of primary EMPD. Positivity for CK20 together with lack of expression of CK7 and TRPS1 favors colonic secondary EMPD, whereas CK7 and CK20 positivity with TRPS1 negativity warrants second-tier testing with CDX2. CDX2 positivity supports colonic origin; however, SATB2-positivity is needed for confirmation since CDX2-positivity can be seen in 20% of urothelial secondary EMPD. Cases showing CK7 positivity with CK20 and TRPS1 negativity should also proceed to the third-tier panel, consisting of p63, GATA3, and uroplakin II/III, to confirm urothelial origin. NKX3.1 represents a promising marker that may aid in identifying prostatic secondary EMPD, though further validation is required. Finally, lesions that are p63-negative with variable GATA3 expression, particularly in the perianal region, may represent primary EMPD. This IHC algorithm allows pathologists to distinguish primary EMPD from secondary EMPD and guide clinical management.

## Figures and Tables

**Table 1:** Clinical characteristics of 12 institutional secondary extramammary Paget disease (EMPD) cases.

Case	Age/Sex (Years)	EMPD Location	Primary Cancer	Duration Between EMPD and Primary Malignancy (Months)	Follow-Up Duration (Months)	Recurrence	Survival Status
1	66/F	Vulva, perineum, anal/perianal	Colorectal	84, before	171	yes	dead
2	70/M	Anal/perianal	Colorectal	84, before	99	yes	alive
3	68/F	Vulva	Colorectal	15, after	33	yes	alive
4	90/M	Anal/perianal	Colorectal	0 (concurrent)	0	NA	dead
5	77/F	Anal/perianal	Colorectal	2, before	26	no	alive
6	83/F	Anal/perianal	Colorectal	5, before	41	no	alive
7	61/M	Anal/perianal	Colorectal	4, before	50	no	dead
8	64/M	Perineum	Urothelial	24, after	97	no	alive
9	69/M	Penis	Urothelial	0 (concurrent)	159	yes	alive
10	70/M	Penis	Urothelial	38, after	12	yes	dead
11	62/F	Stoma	Urothelial	47, after	146	no	alive
12	83/M	Lower abdomen	Urothelial	109, after	21	yes	dead

F: female, M: male, NA: not available.

**Table 2:** Summary of immunohistochemical findings of 12 institutional secondary extramammary Paget disease cases.

Case	Primary Cancer	CDX2	CK20	CK7	GATA3	GCDFP15	p63	SATB2	TRPS1	Uroplakin II/III
1	Colorectal	ND	pos	neg	ND	ND	ND	ND	ND	ND
2	Colorectal	ND	pos	pos	ND	ND	ND	ND	ND	ND
3	Colorectal	pos	pos	pos	ND	ND	ND	ND	ND	ND
4	Colorectal	pos	pos	neg	ND	neg	ND	ND	ND	ND
5	Colorectal	pos	pos	pos	ND	neg	neg	pos	neg	ND
6	Colorectal	pos	pos	pos	neg	neg	neg	pos	neg	ND
7	Colorectal	pos	pos	pos	ND	neg	neg	neg	neg	ND
8	Urothelial	pos	pos	pos	pos	neg	pos	neg	neg	pos
9	Urothelial	neg	pos	pos	pos	neg	pos	neg	neg	ND
10	Urothelial	pos	pos	pos	pos	neg	pos	neg	neg	ND
11	Urothelial	neg	pos	pos	ND	ND	neg	neg	neg	ND
12	Urothelial	pos	pos	pos	ND	neg	pos	neg	neg	ND
	Total, N **%**	8/10 80%	12/12 100%	10/12 83.3%	3/4 75%	0/8 0%	4/8 50%	2/8 25%	0/8 0%	1/1 100%

Pos: positive, neg: negative, ND: not done.

**Table 3:** Summary of immunostaining results of 480 primary (published cases) versus 132 secondary (120 published cases and 12 in current series) extramammary Paget disease cases.

Stain	Primary, N = 480	Secondary				*p*-Value ^§^ (Overall Fisher’s Exact Test)
		Colonic N = 86	Urothelial N = 41	Prostatic N = 5	Total Secondary N = 132	
**CDX2**	9/104 (9%)	51/53 (96%)	3/15 (20%)	0/3 (0%)	54/71 (76%)	<0.001 *
**CK7**	213/217 (98%)	50/70 (71%)	34/34 (100%)	3/3 (100%)	87/107 (81%)	<0.001 *
**CK20**	46/209 (22%)	63/71 (89%)	30/36 (83%)	0/3 (0%)	93/110 (85%)	<0.001 *
**GATA3**	14/14 (100%)	1/7 (14%)	10/10 (100%)	NA	11/17 (65%)	<0.001 *
**GCDFP15**	120/177 (68%)	0/56 (0%)	3/20 (15%)	1/3 (33%)	4/79 (5%)	<0.001 *
**NKX3.1**	NA	NA	0/2 (0%)	NA	0/2 (0%)	NA
**p63**	1/69 (1%)	0/3 (0%)	12/13 (92%)	NA	12/16 (75%)	<0.001 *
**PSA**	22/49 (45%)	NA	NA	2/2 (100%)	2/2 (100%)	0.216
**SATB2**	1/7 (14%)	10/12 (83%)	0/5 (0%)	NA	10/17 (59%)	0.001 *
**TRPS1**	91/101 (90%)	0/14 (0%)	0/8 (0%)	NA	0/22 (0%)	<0.001 *
**Uroplakin II/III**	0/14 (0%)	NA	5/7 (71%)	NA	5/7 (71%)	0.001 *
Cam 5.2	25/25 (100%)	NA	2/2 (100%)	NA	2/2 (100%)	
CEA	77/93 (83%)	15/17 (88%)	1/11 (9%)	0/1 (0%)	16/29 (55%)	
EMA	56/56 (100%)	2/2 (100%)	2/2 (100%)	NA	4/4 (100%)	
HER2	41/66 (62.1%)	0/9 (0%)	7/10 (70%)	3/3 (100%)	10/22 (45%)	
p40	0/3 (0%)	0/5 (0%)	NA	NA	0/5 (0%)	
B72.3	21/22 (95%)	NA	NA	NA	NA	
CD15	0/2 (0%)	0/3 (0%)	NA	NA	0/3 (0%)	
CK1	0/19 (0%)	0/5 (0%)	0/2 (0%)	NA	0/7 (0%)	
CK1,5,10,14	0/19 (0%)	0/5 (0%)	0/2 (0%)	NA	0/7 (0%)	
CK10	0/19 (0%)	0/8 (0%)	0/2 (0%)	NA	0/10 (0%)	
CK13	0/19 (0%)	0/8 (0%)	0/2 (0%)	NA	0/10 (0%)	
CK15	0/16 (0%)	NA	NA	NA	NA	
CK18	not done	NA	1/1 (100%)	NA	1/1 (100%)	
CK19	16/16 (100%)	NA	1/1 (100%)	NA	1/1 (100%)	
CK5/6	0/10 (0%)	0/1 (0%)	NA	NA	0/1 (0%)	
CK8	not done	4/4 (100%)	1/1 (100%)	NA	5/5 (100%)	
Cyclin D1	36/43 (84%)	3/6 (50%)	2/6 (33%)	3/3 (100%)	8/15 (53%)	
ER	0/42 (0%)	0/1 (0%)	0/1 (0%)	NA	0/2 (0%)	
Lysozyme	0/2 (0%)	0/3 (0%)	NA	NA	0/3 (0%)	
MUC1	3/3 (100%)	2/6 (33%)	NA	NA	2/6 (33%)	
MUC2	3/3 (100%)	6/7 (96%)	NA	NA	6/7 (96%)	
MUC5AC	NA	0/1 (0%)	NA	NA	0/1 (0%)	
MUC6	NA	0/1 (0%)	NA	NA	0/1 (0%)	
p16	not done	NA	6/8 (75%)	NA	6/8 (75%)	
P501S	0/16 (0%)	NA	NA	NA	NA	
p53	16/20 (80%)	NA	0/1 (0%)	NA	0/1 (0%)	
Pan-keratin	NA	3/3 (100%)	NA	NA	3/3 (100%)	
PD-L1	3/6 (50%)	NA	NA	NA	NA	
PR	not done	NA	0/1 (0%)	NA	0/1 (0%)	
RANKL	6/6 (100%)	NA	NA	NA	NA	
S100	0/22 (0%)	0/1 (0%)	0/2 (0%)	NA	0/3 (0%)	
Thrombomodulin	NA	NA	1/1 (100%)	NA	1/1 (100%)	
WT1	NA	NA	0/1 (0%)	NA	0/1 (0%)	
References	[13,14,15,16,17,18,19,20,21,22,23,24,25,26,27,28,29,30,31,32,33,34,35,36,37]	[13,14,16,19,20,24,25,26,27,28,29,31,38,39,40,41,42,43,44]	[13,14,18,19,21,23,29,38,43,45,46,47,48,49,50,51,52,53,54,55]	[29,30,56,57]		

^§^ Overall Fisher’s exact across primary, colonic, urothelial, and prostatic EMPD. * *p* < 0.05, statistically significant. NA: not available.

**Table 4:** Pairwise comparison of immunohistochemical positivity between subgroups.

Immunostain	Bonferroni Adjusted *p*-Value					
	Primary vs. Colonic	Primary vs. Urothelial	Primary vs. Prostatic	Colonic vs. Urothelial	Colonic vs. Prostatic	Urothelial vs. Prostatic
CDX2	<0.001 *	1	1	<0.001 *	<0.001 *	1
CK7	<0.001 *	1	1	<0.001 *	1	-
CK20	<0.001 *	<0.001 *	1	1	<0.018 *	0.054
GATA3	<0.001 *	-	NA	0.003 *	NA	NA
GCDFP15	<0.001 *	<0.001 *	1	0.096	0.306	1
p63	1	<0.001 *	NA	0.042 *	NA	NA
PSA	NA	NA	0.216	NA	NA	NA
SATB2	0.036 *	NA	NA	0.018 *	NA	NA
TRPS1	<0.001 *	<0.001 *	NA	-	NA	NA
Uroplakin II/III	NA	0.001 *	NA	NA	NA	NA

* *p* < 0.05, statistically significant, NA: not available.

## Data Availability

All data supporting the findings of this study are available within the paper and Supplementary Information.

## Supplementary Materials

The following supporting information can be downloaded at https://www.mdpi.com/article/10.3390/cancers17244014/s1, Table S1: Percentage of IHC positivity in primary versus secondary published cohort and current cohort. Figure S1: Expanded immunohistochemical diagnostic algorithm for primary and secondary extramammary Paget disease. Detailed immunohistochemical diagnostic algorithm for extramammary Paget disease (EMPD). This expanded flow chart illustrates the stepwise three-tier evaluation incorporating CK7, CK20, and TRPS1 as the initial screening panel, followed by CDX2 and SATB2 for further stratification, and p63, GATA3, uroplakin II/III, PSA, and NKX3.1 as tertiary markers. The algorithm outlines both the most likely diagnostic pathways—distinguishing primary EMPD from colonic, urothelial, and prostatic secondary EMPD—and the alternative pathways for cases with ambiguous, discordant, or partially overlapping immunoprofiles.

## Abbreviations

The following abbreviations are used in this manuscript:

CDX2	Caudal-type homeobox 2
CK	Keratin
EMBASE	Excerpta Medica dataBASE
EMPD	Extramammary Paget disease
GATA3	GATA binding protein 3
GCDFP15	Gross cystic disease fluid protein 15
IQR	Interquartile range
MUC	Mucin
NKX3.1	NK3 Homeobox 1
PDL1	Programmed death-ligand 1
PR	Progesterone receptor
PSA	Prostatic-specific antigen
STROBE	Strengthening the Reporting of Observational Studies in Epidemiology
WT1	Wilm’s tumor 1
SATB2	Special AT-rich sequence-binding protein 2
SD	Standard deviation
TRPS1	Trichorhinophalangeal syndrome type 1
