# Supplementary material for: Diagnostic Algorithm for Secondary Extramammary Paget Disease from Institutional Cases and Literature Review
Source: Cancers (Basel). 2025 Dec 17;17(24):4014. doi: 10.3390/cancers17244014 (PMC12730616; doi:10.3390/cancers17244014)
Supplement: Supplementary file 1 [file cancers-17-04014-s001.zip › Supplementary Figure S1.pdf]

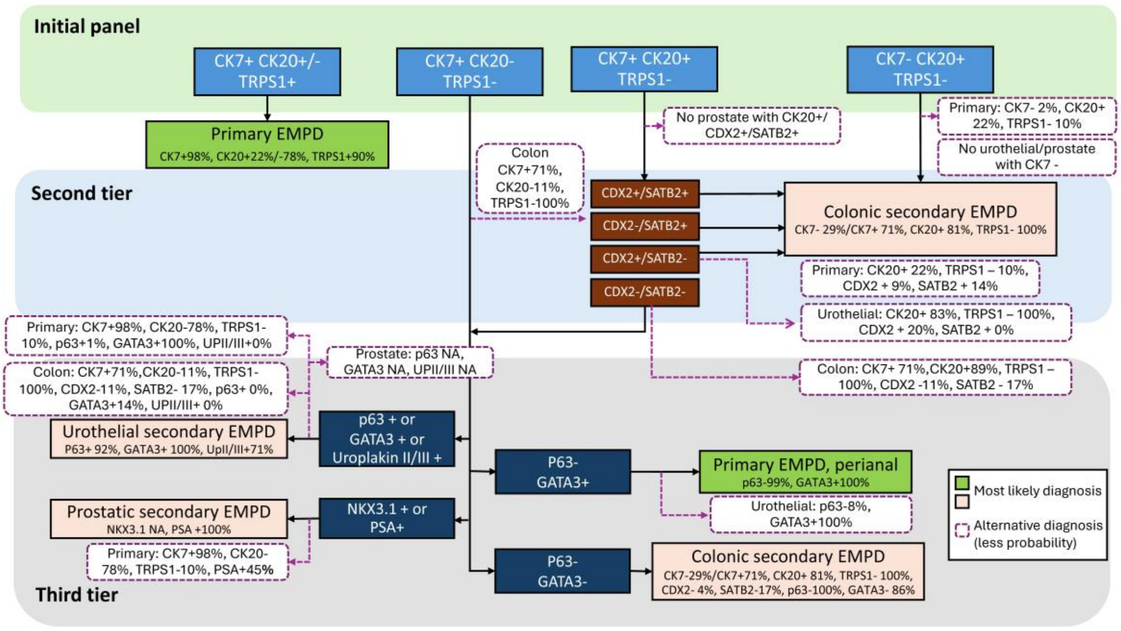

Supplementary Figure S1. Expanded immunohistochemical diagnostic algorithm for primary and secondary extramammary Paget disease. Detailed immunohistochemical diagnostic algorithm for extramammary Paget disease (EMPD). This expanded flow chart illustrates the stepwise three-tier evaluation incorporating CK7, CK20, and TRPS1 as the initial screening panel, followed by CDX2 and SATB2 for further stratification, and p63, GATA3, uroplakin II/III, PSA, and NKX3.1 as tertiary markers. The algorithm outlines both the most likely diagnostic pathways—distinguishing primary EMPD from colonic, urothelial, and prostatic secondary EMPD—and the alternative pathways for cases with ambiguous, discordant, or partially overlapping immunoprofiles.
