# Supplementary material for: Diagnostic Algorithm for Secondary Extramammary Paget Disease from Institutional Cases and Literature Review
Source: Cancers (Basel). 2025 Dec 17;17(24):4014. doi: 10.3390/cancers17244014 (PMC12730616; doi:10.3390/cancers17244014)
Supplement: Supplementary file 1 [file cancers-17-04014-s001.zip › Supplementary Table S1.pdf]

Supplementary Table S1. Percentage of IHC positivity in primary versus secondary published cohort and current cohort.

Stain	Primary, N (%) 480	Secondary, N (%)								
		Literature review (N=120)			Current cohort (N=12)		Total cases (N=132)			
		Colonic (N=79)	Urothelial (N=36)	Prostatic (N=5)	Colonic (N=7)	Urothelial (N=5)	Colonic (N=86)	Urothelial (N=41)	Prostatic (N=5)	Total (N=132)
AR	24/72 (33.3%)	0/6 (0%)	1/7 (14.3%)	0/3 (0%)	NA	1/1 (100%)	0/6 (0%)	2/8 (25%)	0/3 (0%)	2/17 (12%)
Cam 5.2	25/25 (100%)	NA	2/2 (100%)	not done	NA	NA	NA	2/2 (100%)	NA	2/2 (100%)
CDX2	9/104 (9%)	46/48 (96%)	0/10 (0%)	0/3 (0%)	5/5 (100%)	3/5 (60%)	51/53 (96%)	3/15 (20%)	0/3 (0%)	54/71 (76%)
CEA	77/93 (83%)	14/16 (88%)	1/11 (9%)	0/1 (0%)	1/1 (100%)	NA	15/17 (88%)	1/11 (9%)	0/1 (0%)	16/29 (55%)
CK20	46/209 (22%)	56/64 (88%)	25/31 (81%)	0/3 (0%)	7/7 (100%)	5/5 (100%)	63/71 (89%)	30/36 (83%)	0/3 (0%)	93/110 (85%)
CK7	213/217 (98%)	45/63 (71%)	29/29 (100%)	3/3 (100%)	5/7 (71%)	5/5 (100%)	50/70 (71%)	34/34 (100%)	3/3 (100%)	87/107 (81%)
EMA	56/56 (100%)	2/2 (100%)	2/2 (100%)	NA	NA	NA	2/2 (100%)	2/2 (100%)	NA	4/4 (100%)
GATA3	14/14 (100%)	1/6 (16.7%)	7/7 (100%)	NA	0/1 (0%)	3/3 (100%)	1/7 (14.3%)	10/10 (100%)	NA	11/17 (65%)
GCDFP15	120/177 (68%)	0/52 (0%)	3/16 (19%)	1/3 (33%)	0/4 (0%)	0/4 (0%)	0/56 (0%)	3/20 (15%)	1/3 (33%)	4/79 (5%)
HER2	41/66 (62%)	0/9 (0%)	7/10 (70%)	3/3 (100%)	NA	NA	0/9 (0%)	7/10 (70%)	3/3 (100%)	10/22 (45%)
NKX3.1	NA	NA	NA	NA	NA	0/2 (0%)	NA	0/2 (0%)	NA	0/2 (0%)
P40	0/3 (0%)	0/5 (0%)	NA	NA	NA	NA	0/5 (0%)	NA	NA	0/5 (0%)
P63	1/69 (1%)	NA	8/8 (100%)	NA	0/3 (0%)	4/5 (80%)	0/3 (0%)	12/13 (92.3%)	NA	12/16 (75%)
PSA	22/49 (45%)	NA	NA	2/2 (100%)	NA	NA	NA	NA	2/2 (100%)	2/2 (100%)
SATB2	1/7 (14%)	8/9 (89%)	NA	NA	2/3 (67%)	0/5 (0%)	10/12 (83%)	0/5 (0%)	NA	10/17 (59%)
TRPS1	91/101 (90%)	0/11 (0%)	0/3 (0%)	NA	0/3 (0%)	0/5 (0%)	0/14 (0%)	0/8 (0%)	NA	0/22 (0%)
Uroplakin II/III	0/14 (0%)	NA	4/6 (67%)	NA	NA	1/1 (100%)	NA	5/7 (71%)	NA	5/7 (71%)
B72.3	21/22 (95%)	NA	NA	NA	NA	NA	NA	NA	NA	NA
CD15	0/2 (0%)	0/3 (0%)	NA	NA	NA	NA	0/3 (0%)	NA	NA	0/3 (0%)
CK1	0/19 (0%)	0/5 (0%)	0/2 (0%)	NA	NA	NA	0/5 (0%)	0/2 (0%)	NA	0/7 (0%)
CK1,5,10,14	0/19 (0%)	0/5 (0%)	0/2 (0%)	NA	NA	NA	0/5 (0%)	0/2 (0%)	NA	0/7 (0%)
CK10	0/19 (0%)	0/8 (0%)	0/2 (0%)	NA	NA	NA	0/8 (0%)	0/2 (0%)	NA	0/10 (0%)
CK13	0/19 (0%)	0/8 (0%)	0/2 (0%)	NA	NA	NA	0/8 (0%)	0/2 (0%)	NA	0/10 (0%)
CK15	0/16 (0%)	NA	NA	NA	NA	NA	NA	NA	NA	NA

Stain	Primary, N (%) 480	Secondary, N (%)								
		Literature review (N=120)			Current cohort (N=12)		Total cases (N=132)			
		Colonic (N=79)	Urothelial (N=36)	Prostatic (N=5)	Colonic (N=7)	Urothelial (N=5)	Colonic (N=86)	Urothelial (N=41)	Prostatic (N=5)	Total (N=132)
CK18	NA	NA	1/1 (100%)	NA	NA	NA	NA	1/1 (100%)	NA	1/1 (100%)
CK19	16/16 (100%)	NA	1/1 (100%)	NA	NA	NA	NA	1/1 (100%)	NA	1/1 (100%)
CK5/6	0/10 (0%)	0/1 (0%)	NA	NA	NA	NA	0/1 (0%)	NA	NA	0/1 (0%)
CK8	NA	3/3 (100%)	1/1 (100%)	NA	1/1 (100%)	NA	4/4 (100%)	1/1 (100%)	NA	5/5 (100%)
Cyclin D1	36/43 (84%)	3/6 (50%)	2/6 (33%)	3/3 (100%)	NA	NA	3/6 (50%)	2/6 (33%)	3/3 (100%)	8/15 (53%)
ER	0/42 (0%)	0/1 (0%)	0/1 (0%)	NA	NA	NA	0/1 (0%)	0/1 (0%)	NA	0/2 (0%)
Lysozyme	0/2 (0%)	0/3 (0%)	NA	NA	NA	NA	0/3 (0%)	NA	NA	0/3 (0%)
MUC1	3/3 (100%)	2/5 (40%)	NA	NA	0/1 (0%)	NA	2/6 (33%)	NA	NA	2/6 (33%)
MUC2	3/3 (100%)	6/6 (100%)	NA	NA	0/1 (0%)	NA	6/7 (96%)	NA	NA	6/7 (96%)
MUC5AC	NA	NA	NA	NA	0/1 (0%)	NA	0/1 (0%)	NA	NA	0/1 (0%)
MUC6	NA	NA	NA	NA	0/1 (0%)	NA	0/1 (0%)	NA	NA	0/1 (0%)
P16	NA	NA	6/8 (75%)	NA	NA	NA	NA	6/8 (75%)	NA	6/8 (75%)
P501S	0/16 (0%)	NA	NA	NA	NA	NA	NA	NA	NA	NA
P53	16/20 (80%)	NA	0/1 (0%)	NA	NA	NA	NA	0/1 (0%)	NA	0/1 (0%)
Pankeratin	NA	3/3 (100%)	NA	NA	NA	NA	3/3 (100%)	NA	NA	3/3 (100%)
PD-L1	3/6 (50%)	NA	NA	NA	NA	NA	NA	NA	NA	NA
PR	NA	NA	0/1 (0%)	NA	NA	NA	NA	0/1 (0%)	NA	0/1 (0%)
RANKL	6/6 (100%)	NA	NA	NA	NA	NA	NA	NA	NA	NA
S100	0/22 (0%)	0/1 (0%)	0/2 (0%)	NA	NA	NA	0/1 (0%)	0/2 (0%)	NA	0/3 (0%)
Thrombomodulin	NA	NA	1/1 (100%)	NA	NA	NA	NA	1/1 (100%)	NA	1/1 (100%)
WT1	NA	NA	0/1 (0%)	NA	NA	NA	NA	0/1 (0%)	NA	0/1 (0%)
